# Supplementary material for: Knowledge, attitudes and practices toward female genital schistosomiasis among community women and healthcare professionals in Kimpese region, Democratic Republic of Congo
Source: PLoS Negl Trop Dis. 2024 Jul 12;18(7):e0011530. doi: 10.1371/journal.pntd.0011530 (PMC11268635; doi:10.1371/journal.pntd.0011530)
Supplement: S1 File — Text A: FGS KAP Survey questionnaire for the community women in Kifua II. Text B: FGS KAP Survey questionnaire for the healthcare professionals in Kimpese Health Zone. Text C: Summary of the sociodemographic characteristics of community women and healthcare professionals. Text D: Summary of the healthcare professionals’ perception, practices, and management of female genital schistosomiasis in Kimpese Health Zone. Text E: Summary of FGS-like signs and symptoms experienced by the community women at Kifua II. (DOCX) [file pntd.0011530.s001.docx]

**Knowledge, attitudes and practices toward female genital schistosomiasis among community women and healthcare professionals in Kimpese region, Democratic Republic of Congo**

**Text A**

## FGS KAP Survey questionnaire for the community women

**Instructions to the interviewer:**

It is important to begin with a short conversation with the respondent before starting to ask the questions. This initial conversation should be the same for all the respondents to ensure that they all are at the same level of understanding prior to the start of the interview.

Consider the following:

1. Introduce yourself, welcome the respondent and thank them for finding the time to participate in the survey
2. Explain the reason for conducting the survey: research study on **“knowledge, attitudes and practices towards female genital schistosomiasis (FGS)”**
3. Assure them on resourcefulness of their contribution, guarantee confidentiality and that the information they provide will be treated anonymously and only used for the study.
4. Give them a chance to ask any question regarding the research before beginning the interview.

Interview Date: __ __ /__ __ /__ __ __ __ (DD/MM/YYYY)

Respondent code: _________ (for privacy and confidentiality of information/data)

Interviewer Name: ________________________________

Location: _____________________________

1. **DEMOGRAPHIC INFORMATION**
2. **What is your age/day of birth? _______________________**
3. **What is your marital status?**
4. Single
5. Married in a monogamous marriage
6. Married in a polygamous marriage
7. Divorced
8. Widowed
9. Separated
10. Cohabiting (no documentation)
11. **If you are single or divorced or separated, is this due to health reasons?**
    1. Yes
    2. No
    3. Other
12. **What is your highest level of education?**
    1. Without education
    2. Primary school finished
    3. Primary school not finished
    4. Secondary school finished
    5. Secondary school not finished
    6. Higher level
13. **If without education, are you able to read and write?**
    1. Yes
    2. No
14. **What is your employment status?**
    1. Unemployed
    2. Self-employed
    3. Employed
    4. Student
    5. Retired
15. **If self-employed, in which sector?**
16. Farming
17. Livestock rearing
18. Fishing/fish trading
19. Food and beverage vending (market/restaurant)
20. Shopkeeper/fashion (boutique/tailoring)
21. Sex profession
22. Other: Specify ____________________
23. **If employed, in which sector?**
    1. Teacher
    2. Politician: Specify which function______________________________?
    3. Public service: Specify which__________________________________?
    4. Private sector: Specify which__________________________________?
    5. Religious leader: Specify which function_________________________?
    6. Informal employment (neither taxed nor monitored by any form of government): Specify which______________________________?
24. **How much do you earn per month?**
25. Less than 100,000 CDF
26. 100,000 - 199,000 CDF
27. 200,000 - 299,000 CDF
28. 300,000 - 399,000 CDF
29. More than 400,000 CDF
30. **How far is the nearest health facility in the area?**
31. 0–5 kilometres
32. 6-10 kilometres
33. 11–15 kilometres
34. More than 15 kilometres
35. **KNOWLEDGE**
36. **Do you know what bilharzia/bilari is?**
    1. Yes *(if yes, go to the question 15, what FGS is.)*
    2. No
    3. Not sure

If “No or Not sure” check the brief explanation of bilharzia (bilari) below:

*Bilharzia (bilari) is an infection caused by parasitic worms called schistosomes. Some of the symptoms include abdominal pain, diarrhea, blood in stool and urine, severe fever, belly enlargement, skin rush, stunted growth and reduced learning ability.*

1. **Do you or a member of your family or friends experienced the following symptoms:**

Abdominal pain, diarrhoea, tired, blood present in stool or urine, belly enlargement, (severe) fever, skin rash, stunted growth, reduced learning ability?

- 1. Yes
  2. No *(stop here for the knowledge section and provide an information leaflet about bilari and bilari ya banketo)*

1. **How are these symptoms treated?**
2. **Have you ever heard of female genital schistosomiasis heard of (FGS) (bilari ya banketo)?**
3. Yes *(Skip the next question)*
4. No
5. Not sure

If “No or Not sure” check the brief explanation of FGS (bilari ya banketo) below:

*FGS (Bilari ya banketo) is a form of bilharzia (bilari) that affects the genital areas of an infected individual. Some of the symptoms include bleeding and pain during intercourse, wounds on the vagina or cervix, abnormal vaginal discharge with smell and coloured, genital itching and contact bleeding during an examination. FGS can also have long term effects such as reduced fertility, miscarriage, and increased risks to HIV.*

1. **Do you now know what FGS (bilari ya banketo) is?**
   1. Yes
   2. No *(If she still don’t know after the explanation, provide an information leaflet about bilari ya banketo and end the Knowledge section here*)
2. **Where did you first hear about FGS (bilari ya banketo)?** *(Tick only one option)*
   1. Radio
   2. Television
   3. Billboards
   4. Newspapers and magazines, brochures, posters and other printed materials
   5. Healthcare workers/ Treatment campaigns
   6. Community meeting/ Religious leaders/ Political leaders
   7. Family, friends, neighbours and colleagues
   8. I do not remember
   9. Other (please specify)
3. **According to you, what do you think FGS (bilari ya banketo) is?** *(Tick only one option)*
   1. It is a disease
   2. It is a curse
   3. I don’t remember
   4. Other: Specify ____________________
4. **What are the symptoms of FGS (bilari ya banketo)?** *(Tick all that apply) (If she doesn’t understand the symptoms, provide a brief explanation to her)*
   1. Low abdominal pain
   2. Irregular menstruation
   3. Contact bleeding (bleeding during examination)
   4. Unusual vaginal discharge (grey, white, yellow, or smelly)
   5. Swelling or growths on vulva, vagina, cervix
   6. Bleeding or spotting after intercourse
   7. Pain during or after intercourse (Dyspareunia)
   8. Genital itching or burning sensation
   9. Painful and heavy bleeding during menstruation
   10. Other Specify ____________________
5. **What are the complications of FGS (bilari ya banketo)?** *(Tick all that apply) (Provide an explanation about the complications if she doesn’t understand)*
   1. Reduced fertility
   2. Ectopic pregnancy
   3. Delayed puberty
   4. Miscarriage
   5. Early labor
   6. Early menopause
   7. Vaginal itching and wounds
   8. Swelling of the lower part of the uterus
   9. Involuntary urination when coughing, laughing or jumping
   10. Increased risk of HIV, HPV (human papilloma virus) infection and cervical cancer
   11. Other: Specify _____________________
6. **How is FGS (bilari ya banketo) transmitted?** *(Tick all responses that apply) (Indicate that the transmission is similar to that of schistosomiasis (bilari))*
7. Handshakes
8. Drinking untreated water
9. Swimming, bathing, baptism in river, streams, ponds
10. Washing clothes/Utensils in river, streams, ponds
11. Playing with soil
12. Eating unwashed fruits and vegetables
13. Working in irrigation/agriculture fields
14. Fishing
15. Witchcraft
16. Sexual contact
17. Bad luck
18. Do not know
19. Other Specify ____________________
20. **Which organisms are responsible for the FGS (bilari ya banketo)?**
21. Mosquitoes
22. Snails
23. Flies
24. Worms
25. Bacteria
26. I don’t know
27. Other Specify ____________________
28. **Who is at risk of contracting FGS (bilari ya banketo)?** *(Tick all that apply)*
29. Married women only
30. Women or girls that are sexually active only
31. Women that cheat on their partners
32. Young girls only
33. All women and girls
34. All boys and men
35. Men or boys who are sexually active
36. Other Specify ____________________
37. **Do you think if infertility, vaginal bleeding, painful menstruation and genital itching symptoms of FGS (bilari ya banketo) can be treated?**
38. Yes
39. No
40. Not sure
41. **If yes, how can someone with these symptoms of FGS (bilari ya banketo) be treated?** *(Tick all that apply)*
    1. Praying
    2. Home rest without medicine
    3. Praziquantel (Biltricide)
    4. Drugs given by health centre/pharmacy/ over the counter
    5. Traditional/herbal medicines
    6. Rituals
    7. I don’t know
    8. Other: Specify _______________________
42. **How can FGS (bilari ya banketo) be prevented and controlled?** *(Tick all responses that apply)*
    1. TreatKment for all infected persons
    2. Avoid contact of water
    3. Avoid open defecation/Urination
    4. Wash your hands after visiting the toilet
    5. Avoid uncooked meat, and unwashed fruits and vegetables
    6. Avoid walking barefoot and playing in the soil
    7. Health education
    8. Snail control
    9. Don’t know
43. **How can an infected person contribute towards spreading FGS (bilari ya banketo)?**
    1. Urinating/ defecating by water
    2. Physical contact with an uninfected person
    3. Sexual contact
    4. Don’t know
    5. Other Specify ____________________
44. **ATTITUDES**

***Now I would like you to give your opinion on female genital schistosomiasis (FGS) by stating the extent to which you agree or disagree with each of the following statements. The options range from Strongly Disagree (1), Disagree (2), Not Sure (3), Agree (4), and Strongly Agree (5)***

| **#** | **Statement** | **1** | **2** | **3** | **4** | **5** |
| --- | --- | --- | --- | --- | --- | --- |
| **C1.** | FGS (bilari ya banketo) is a very serious disease |  |  |  |  |  |
| **C2.** | It is necessary to take preventive measures against FGS (bilari ya banketo) |  |  |  |  |  |
| **C3.** | It is my responsibility to prevent infection from FGS (bilari ya banketo) |  |  |  |  |  |
| **C4.** | It is important to know whether I have FGS (bilari ya banketo) or not |  |  |  |  |  |
| **C5.** | I feel comfortable in providing my stool and urine sample for diagnosis |  |  |  |  |  |
| **C6** | I feel comfortable with a gynaecological examination |  |  |  |  |  |
| **C7.** | It is important to avoid contact with water |  |  |  |  |  |
| **C8.** | Defecating and urinating in the toilet is important for my health |  |  |  |  |  |
| **C9.** | An infected person contributes towards spreading FGS (bilari ya banketo) |  |  |  |  |  |
| **C10.** | FGS (bilari ya banketo) can be treated |  |  |  |  |  |
| **C11.** | Taking medication for FGS (bilari ya banketo) is important for my health |  |  |  |  |  |
| **C12.** | My husband will leave me if I have FGS (bilari ya banketo) /if I am infertile |  |  |  |  |  |
| **C13** | My husband’s family and my community will not respect me if I am infertile or have FGS (bilari ya banketo) |  |  |  |  |  |
| **C14.** | I can talk/I talk to my husband about FGS (bilari ya banketo) symptoms |  |  |  |  |  |
| **C15.** | I talk with my relatives about FGS (bilari ya banketo) |  |  |  |  |  |
| **C16.** | I would take action if I found that I had FGS (bilari ya banketo) by going to the hospital |  |  |  |  |  |
| **C17.** | If I find that I have FGS (bilari ya banketo), I should see a traditional health practitioner |  |  |  |  |  |
| **C18.** | If I find that I have FGS (bilari ya banketo), I should see a religious leader |  |  |  |  |  |
| **C19.** | People who have FGS (bilari ya banketo) are cursed |  |  |  |  |  |
| **C20.** | It is important for me to be informed about FGS (bilari ya banketo) |  |  |  |  |  |

***NB: For respondents whose answer to question 16 was NO (on what FGS is), use the attitude information at the end of the questionnaire.***

1. **PRACTICES**

**D1. HOUSEHOLD ACTIVITIES AND OCCUPATIONAL RISK**

1. **What are the main sources of water used in your household?**
   1. Piped water
   2. Unprotected well
   3. Protected well
   4. Bore hole
   5. River /stream/lake/pond/irrigation canal/Fall/cascade
   6. Rain water
   7. Water truck / water vendor
   8. Standpost (Stanpipe)
   9. Spring/Source
2. **What are the activities that require you or your household members to be in contact with water?**
   1. Fetching water
   2. Bathing
   3. Swimming
   4. Baptism
   5. Laundry
   6. Fishing
   7. Agriculture
   8. Retting of cassava dishes
   9. Taking animals for drinking
   10. None
   11. Other Specify ____________________
3. **How long do you or your household member need to undertake these activities in contact with water?**
   1. Less than 5 minutes
   2. 5 to 10 minutes
   3. 10 to 15 minutes
   4. More than 15 minutes
4. **How many times a day do you or your family members undertake these activities in contact with water?**
5. Once
6. Twice
7. Thrice
8. More than 3 times

**D2. SANITATION AND HYGIENE**

1. **What kind of toilet does your household use?**
   1. Communal latrine
   2. Flush system
   3. Pit latrine
   4. None
   5. Other Specify ____________________
2. **Have you ever defecated/urinated outside the toilet?** *(If response is no skip the next two questions)*
3. Yes
4. No
5. **Where exactly did you defecate/urinate outside the toilet?**
   1. In an open space
   2. In the water/close to water body
   3. In the bush
   4. In a bucket/tin
   5. I don’t remember
   6. Other Specify ____________________
6. **If yes, why did you not use the toilet?**
   1. No access to toilets
   2. Toilets are there but poorly maintained
   3. The toilets were busy/crowded
   4. I do not like to use the toilet
   5. My religion/culture does not allow me
   6. Lack of water to flush toilets
   7. Natures call
   8. Other Specify ____________________
7. **How often do you defecate/urinate outside the toilet?**
   1. Every day and when nature calls
   2. It was only once
   3. Do not remember
   4. Other Specify ____________________

**D3. SYMPTOMS FOR URINARY TRACT SCHISTOSOMIASIS, FGS OR SEXUALLY TRANSMITTED INFECTIONS (STIS)**

1. **Do you see your menses?** []Yes []No *(Skip the next two questions)*
2. **Is your menses painful?** []Always very painful []Sometimes painful []Normal
3. **Is your menses irregular?** []Yes, every month []Not every month []It stopped
4. **Do you have pain when urinating?** [] Yes [] No
5. **Do you have difficulty in urinating (urine not coming out fluently)?** []Yes []No
6. **Do you have sudden uncontrollable and unexpected urge to urinate and mostly cannot hold it? Even when you cough it comes out?** []Yes []No
7. **Do you see blood in your urine?** []Yes []No
8. **If yes, when did you lastly see blood in your urine?** []Always []Sometimes []Once in a while
9. **Do you sometimes have itching in your private part?** []Yes []No (*Skip the next two questions*
10. **If yes, how often do you experience this?** []Once a while []frequently
11. **When was the last time? _______________-**
12. **Do you have a feeling of burning within your private part?** []Yes []No
13. **If yes, how often do you experience this?** []Once a while []Frequently
14. **Do you sense a swelling/lumps within your private part?** []Yes []No []Do not know
15. **Do you have any discharge that comes from your vagina?** []Yes []No *(Skip the next five questions*
16. **Does the discharge have an odour?** []Yes []No
17. **Do you see the colour in the discharge?** []Yes []No
18. **What Color is the discharge?** []White []Grey []Green/Yellow []Brown
19. **Do you think this is normal?** **?** []Yes []No []I do not know
20. **When did you start observing the discharge?** Date ____________
21. **After sexual intercourse do you have a discharge?** []Yes []No
22. **If yes, is the discharge smelly?** []Yes []No []I do not know
23. **After or during sexual intercourse do you have pain?** []Yes []No []I do not know
24. **Do you have a bloody discharge?** []Yes []No []I do not know
25. **Have you had any miscarriages/pregnancies that passed?** []Yes []No []I do not know
26. **If yes, how many miscarriages?** __________________

**D4. PREVENTION AND HEALTH SEEKING BEHAVIOUR**

1. **What have you done for experiencing any of the symptoms above?**
   1. Visited a health facility
   2. Visited a pharmacist/drug vendor
   3. Visited a village health worker/midwife
   4. Visited a religious leader
   5. Visited a traditional healer
   6. I treated myself
   7. I did nothing
   8. Other: Specify__________________________
2. **If no help was sought (did nothing), what was the reason?**
   1. I didn’t know who to consult
   2. Too difficult to get to a health facility
   3. Did not have time/busy
   4. Too expensive to obtain treatment
   5. Unfriendly healthcare workers
   6. Fear of medication
   7. I feel ashamed
   8. Family member/ friend advised against it
   9. Religious leader advised against it
   10. No reason
   11. I never seek for medical help
   12. Other Specify ____________________
3. **Which tests were done?**
   1. Urine test
   2. Stool test
   3. Sexual Transmitted Infections test
   4. Vaginal/Cervical screening
   5. None
   6. Other Specify ____________________
4. **Have you ever received Praziquantel/Biltricide through a treatment campaign?**
5. Yes
6. No *(skip the next question)*
7. Don’t know *(skip the next question)*
8. **When was the last time you took Praziquantel/Biltricide?**
   1. Within the last 6 months
   2. Within the last 2 years
   3. Cannot remember
9. **Why did you not receive Praziquantel/Biltricide?**
   - 1. There was no campaign in my community
     2. Did not know about the campaign
     3. I wasn’t offered the drug/the drugs were not available
     4. I was not eligible
     5. I was not ill
     6. Family member/ neighbor/friend advised against
     7. My religion does not allow
     8. I was scared of side effects
     9. Drugs don’t work
     10. I prefer traditional medicine
     11. Did not trust distributors
     12. No reason
     13. Other Specify ____________________
10. **Would you accept a vaccine against FGS if a vaccine would be available?**
    1. Yes
    2. No *(ask the reason why)_________________*

**E. FGS INFORMATION AND COMMUNICATION**

1. **Do you wish you could get information about FGS?**
   1. Yes
   2. No *(skip to the last question)*
2. **What information would you wish to get about FGS?** *(Tick all that apply)*

What FGS (bihari ya banketo) is

- - 1. Signs, symptoms and transmission
    2. Prevention
    3. Which treatment is appropriate
    4. Diagnosis (where and how is it done)
    5. Cultural beliefs and practices
    6. Risks associated with infection
    7. Where to get information and to go for treatment

1. **What are the common sources of information can most effectively reach you with information on FGS (bilari ya banketo)?**
   - 1. Newspapers, magazines, brochures, posters and other printed materials
     2. Radio or Television
     3. Mobile Phone messages
     4. Billboards/tableaux
     5. Healthcare workers (relais communautaires)
     6. Nurses or doctors
     7. Family, friends, neighbours and colleagues
     8. Religious leaders, Politicians/Community leaders
     9. Theatre plays and famous actors or singers
     10. Teachers
     11. Other Specify ____________________
2. **Do you have any comment or question about what we have discussed?**
3. Yes
4. No

If yes state here

……………………………………………………………………………………………………………………………………………………………………………………………………………. **Thank you for your participation!!!!**

***Attitude section for respondents whose answer to question 16 was NO (on what FGS is). If needed, repeat the symptoms from C1 in the later questions.***

| **#** | **Statement** | **1** | **2** | **3** | **4** | **5** |
| --- | --- | --- | --- | --- | --- | --- |
| **C1.** | Vaginal bleeding during sex, wounds in the vagina, reduced fertility, miscarriages, delayed puberty and early menopause ***(refer to responses on question 19 and 20)*** are very  serious symptoms and complications |  |  |  |  |  |
| **C2.** | It is necessary to take preventive measures against the disease causing these symptoms and complications |  |  |  |  |  |
| **C3.** | It is my responsibility to prevent against these symptoms and complications |  |  |  |  |  |
| **C4.** | It is important to know whether I have the disease causing these symptoms and complications or not |  |  |  |  |  |
| **C5.** | I feel comfortable in providing my stool and urine sample for diagnosis |  |  |  |  |  |
| **C6** | I feel comfortable with a gynaecological examination |  |  |  |  |  |
| **C7.** | It is important to avoid contact with water |  |  |  |  |  |
| **C8.** | Defecating and urinating in the toilet is important for my health |  |  |  |  |  |
| **C9.** | An infected person contributes towards spreading the disease causing these symptoms and complications |  |  |  |  |  |
| **C10.** | These symptoms and complications can be treated |  |  |  |  |  |
| **C11.** | Taking medication for these symptoms and complications is important for my health |  |  |  |  |  |
| **C12.** | My husband will leave me if I have these symptoms and complications |  |  |  |  |  |
| **C13** | My husband’s family and my community will not respect me if I have these symptoms and complications |  |  |  |  |  |
| **C14.** | I can talk/I talk to my husband about these symptoms and complications |  |  |  |  |  |
| **C15.** | I talk with my relatives about these symptoms and complications |  |  |  |  |  |
| **C16.** | I would take action if I found that I had these symptoms and complications by going to the hospital |  |  |  |  |  |
| **C17.** | If I find that I have these symptoms and complications , I should see a traditional health practitioner |  |  |  |  |  |
| **C18.** | If I find that I have these symptoms and complications , I should see a religious leader |  |  |  |  |  |
| **C19.** | People who have these symptoms and complications are cursed |  |  |  |  |  |
| **C20.** | It is important for me to be informed about the disease causing these symptoms and complications |  |  |  |  |  |

##

**Test B**

## FGS KAP survey questionnaire for the healthcare professionals

**Instructions to the interviewer:**

It is important to begin with a short conversation with the respondent before starting to ask the questions. This initial conversation should be the same for all the respondents to ensure that they all are at the same level of understanding prior to the start of the interview.

Consider the following:

1. Introduce yourself, welcome the respondent and thank them for finding the time to participate in the survey
2. Explain the reason for conducting the survey: research study on “**knowledge, attitudes and practices towards female genital schistosomiasis (FGS)”**
3. Assure them the information they provide will be treated with the highest degree of confidentiality and used for Assure them on resourcefulness of their contribution, guarantee confidentiality and that the information they provide will be treated anonymously and only used for the study.
4. Give them a chance to ask any question regarding the research before beginning the interview. Know that they have the right to stop their participation at any time.

Interview Date: __ __ /__ __ /__ __ __ __ (DD/MM/YYYY)

Respondent code: _________ (for privacy and confidentiality of information/data)

Interviewer Name: ________________________________

Location: _____________________________

1. **KNOWLEDGE**

**1. Do you know what female genital schistosomiasis (FGS) (bilari ya banketo) is?**

- 1. Yes *(skip the next question)*
  2. No
  3. Not sure

If “No or Not sure” check the brief explanation of FGS (bilari ya banketo) below:

*FGS (Bilari ya banketo) is a form of bilharzia (bilari) that affects the genital areas of an infected individual. Some of the symptoms include bleeding and pain during intercourse, wounds on the vagina or cervix, abnormal vaginal discharge with smell and coloured, genital itching and contact bleeding during an examination. FGS can also have long term effects such as reduced fertility, miscarriage, and increased risks to HIV.*

**2. Do you now know what FGS (bilari ya banketo) is?**

- 1. Yes
  2. No *(If she still don’t know after the explanation, provide an information leaflet about bilari ya banketo and end the Knowledge section here*)

**3. What are the signs and symptoms of FGS?**

1. Low abdominal pain
2. Irregular menstruation
3. Contact bleeding (bleeding during examination)
4. Unusual vaginal discharge (grey, white, yellow, or smelly)
5. Swelling or growths on vulva, vagina, cervix
6. Bleeding or spotting after intercourse
7. Pain during or after intercourse (Dyspareunia)
8. Genital itching or burning sensation
9. Painful and heavy bleeding during menstruation
10. Other Specify ____________________

**4. What are the complications of FGS?**

1. Primary infertility (infertility for more than 4 years in a woman who had never been pregnant)
2. Secondary infertility (infertility for more than 4 years in a previously fertile woman
3. Abortion or ectopic pregnancy
4. Delayed puberty
5. Miscarriage
6. Early labor
7. Early menopause
8. Vaginal itching and presence of lesions
9. Inflammation of cervix
10. Involuntary urination when coughing, laughing or jumping
11. Increased risk of HIV and HPV (human papilloma virus) infection and cervical cancer
12. Other: Specify _____________________

**5. Which organisms are responsible for the FGS (bilari ya banketo)?**

1. Mosquitoes
2. Snails
3. Flies
4. Worms
5. Bacteria
6. Don’t know
7. Other Specify _______________

**6. How is FGS (bilari ya banketo) transmitted?** *(Tick all responses that apply*

- 1. Handshakes
  2. Drinking untreated water
  3. Swimming, bathing, baptism in river, streams, ponds whose water is contaminated
  4. Fishing, washing clothes/Utensils in river, streams, ponds whose water is contaminated
  5. Playing with soil
  6. Eating unwashed fruits and vegetables
  7. Working in irrigation/agriculture fields containing contaminated water
  8. Witchcraft
  9. Sexual contact
  10. Bad luck
  11. Do not know
  12. Other Specify ____________________

**7. Who is at risk of getting infected?**

1. Married women only
2. Women or girls that are sexually active only
3. Women that cheat on their partners
4. Young girls only
5. All women and girls who come into contact with contaminated water
6. All boys and men
7. Men or boys who are sexually active
8. Other Specify ____________________

**8. Why is the person at risk of contracting FGS**__________________________

**9. What is the diagnostic test for FGS?**

- 1. Urine test (real-time polymerase chain reaction-PCR, microscopy of urine, haematuria, circulating anodic antigen (CAA), and antibodies)
  2. Colposcopy
  3. Sexual Transmitted Infections test
  4. Stool test
  5. Cervicovaginal lavage (CVL) PCR
  6. Blood test

**10. What is the cost of the test?______________________________**

**11. What is the treatment for persons diagnosed with FGS?**

- 1. Praying
  2. Home rest without medicine
  3. Praziquantel (Biltricide)
  4. Drugs given by health centre/pharmacy/ over the counter
  5. Traditional/herbal medicines
  6. Rituals
  7. I don’t know
  8. Other: Specify _______________________

**12. How much does the treatment of FGS cost?**______________________________

**13. How can FGS (bilari ya banketo) be prevented and controlled?** *(Tick all responses that apply)*

- 1. Treatment for all infected persons
  2. Avoid contact of water
  3. Avoid open defecation/Urination
  4. Wash your hands after visiting the toilet
  5. Avoid uncooked meat, and unwashed fruits and vegetables
  6. Avoid walking barefoot and playing in the soil
  7. Health education
  8. Snail control
  9. Don’t know

**14. How can infected person contribute towards spreading FGS (bilari ya banketo)**

1. Urinating/ defecating by water
2. Physical contact with an uninfected person
3. Sexual contact
4. Don’t know
5. Other Specify ____________________
6. **ATTITUDES**

***Now I would like you to give your opinion on female genital schistosomiasis (FGS) by stating the extent to which you agree or disagree with each of the following statements. The options range from Strongly Disagree (1), Disagree (2), Not Sure (3), Agree (4), and Strongly Agree (5)***

| **#** | **Statement** | **1** | **2** | **3** | **4** | **5** |
| --- | --- | --- | --- | --- | --- | --- |
| **a.** | FGS (bilari ya banketo) is a very serious disease |  |  |  |  |  |
| **b.** | It is necessary to advocate for preventive measures against FGS (bilari ya banketo) |  |  |  |  |  |
| **c.** | It is important to know the diagnosis for FGS (bilari ya banketo). |  |  |  |  |  |
| **c.** | It is my responsibility to diagnose FGS (bilari ya banketo). |  |  |  |  |  |
| **f.** | An infected person contributes towards spreading FGS (bilari ya banketo) |  |  |  |  |  |
| **g.** | FGS (bilari ya banketo) can be treated |  |  |  |  |  |
| **h.** | The medication for FGS (bilari ya banketo) is expensive |  |  |  |  |  |
| **j.** | I can talk / I talk with my colleagues and patients about FGS (bilari ya banketo) |  |  |  |  |  |
| **k.** | People who have FGS (bilari ya banketo) are cursed |  |  |  |  |  |
| **l.** | People with FGS are unfaithful |  |  |  |  |  |
| **m.** | It is important for me to be informed about FGS (bilari ya banketo) |  |  |  |  |  |

1. **PERCEPTION, PRACTICES AND MANAGEMENT**

**15. Do you think FGS is prevalent in Kimpese?**

- 1. Yes *(Skip next question)*
  2. No
  3. Not sure

1. **If no and not sure, what is the reason?____________**
2. **Have you had patients with female genital schistosomiasis (FGS)?**
   1. Yes
   2. No *(Skip the next two questions)*
   3. No sure *(Skip the next two questions)*
3. **If yes, how many cases of FGS have you encountered?**
   1. Less than 5 cases
   2. 5-15 cases
   3. 15-25 cases
   4. 25-35 cases
   5. More than 35 cases
4. **How did you diagnose the patients had FGS? ______________**
5. **Are there diagnostic test equipment for FGS available at your healthcare centre?**_
6. **What is the availability of the Praziquantel (PZQ)/Biltricide) at your health care centre?** ________________________________
7. **DEMOGRAPHIC INFORMATION**
8. **What is your age? _________________**
9. **What is your gender?**
10. Male
11. Female
12. Prefer not to say
13. **What is your level of education? ___________________________**
14. **For how long have you been a health care worker?** __________________________
15. **What is your specialty as a health care worker? ___________________________**
16. **COMMUNICATION INFORMATION**
17. **Do you wish you could get information about FGS?**
    1. Yes
    2. No *(skip to the last question)*
18. **What information would you wish to get about FGS?** *(Tick all that apply)*
19. What FGS (bihari ya banketo) is
20. Signs, symptoms and transmission
21. Prevention and vaccination prospects
22. Diagnosis
23. Risks associated with infection
24. Other Specify_________________
25. **What are the common sources of information can most effectively reach you with information on FGS (bilari ya banketo)?**
26. Newspapers, magazines, brochures, posters and other printed materials
27. Health education programs/campaigns
28. Radio or Television
29. Mobile Phone messages
30. Billboards/tableaux
31. Family, friends, neighbours and colleagues
32. Religious leaders, Politicians/Community leaders
33. Theatre plays and famous actors or singers
34. Other Specify ____________________
35. **Do you have any comment or question about what we have discussed?**
36. Yes
37. No

If yes state here

……………………………………………………………………………………………………………

**Thank you for your participation!**

**NB: The page below visually illustrates female genital schistosomiasis symptoms.** <https://apps.who.int/iris/handle/10665/255855>

**Test C**

Summary of the sociodemographic characteristics of community women and healthcare professionals

| **Variable** | **Response** | **Frequency** | | **Percentage** |
| --- | --- | --- | --- | --- |
| **Community women** | | | | |
| Age groups | 15-25 | | 61 | 30.3 |
|  | 26-36 | | 52 | 25.9 |
|  | 37-47 | | 46 | 22.9 |
|  | 48-59 | | 42 | 20.9 |
| Marital Status | Married in a monogamous marriage | | 110 | 54.7 |
|  | Single | | 48 | 23.9 |
|  | Married in a polygamous marriage | | 22 | 10.9 |
|  | Divorced/windowed/separated | | 21 | 10.5 |
| Education level | Without formal education | | 80 | 39.8 |
|  | Primary education | | 108 | 53.7 |
|  | Secondary education and higher education | | 13 | 6.5 |
| Occupation | Farming | | 121 | 60.2 |
|  | Unemployed | | 42 | 20.9 |
|  | Private sector | | 17 | 8.5 |
|  | Self-employed (shopkeeper, fish trading, fashion (boutique/tailoring) | | 13 | 6.5 |
|  | Student | | 6 | 3 |
|  | Teacher and Nursing | | 2 | 1.0 |
| Monthly income | Less than 100,000 | | 121 | 60.2 |
|  | 100,000 - 199,000 | | 24 | 11.9 |
|  | 200,000 - 299,000 | | 5 | 2.5 |
|  | More than 300,000 | | 3 | 1.5 |
|  | No response | | 48 | 23.9 |
| **Healthcare professionals** | | | | |
| Age groups | 29 - 45 | | 38 | 62.3 |
|  | 46 - 60 | | 21 | 34.4 |
|  | 61 - 76 | | 2 | 3.3 |
| Gender | Male | | 52 | 85.2 |
|  | Female | | 9 | 14.8 |
| Duration working as a healthcare worker | 1 - 10 years | | 27 | 44.3 |
|  | 11 - 20 years | | 29 | 47.5 |
|  | 21 -40 years | | 5 | 8.2 |

**Test D**

Summary of the healthcare professionals’ perception, practices, and management of female genital schistosomiasis in Kimpese Health Zone.

| **Variable** | **Response** | **Frequency** | **Percentage** |
| --- | --- | --- | --- |
| Is FGS prevalent in Kimpese | Yes | 47 | 77.0 |
|  | No /Not sure | 14 | 23.0 |
| Had patients with FGS? | Yes | 40 | 65.6 |
|  | No /Not sure | 21 | 34.4 |
| How many? | 15-25 cases | 4 | 6.6 |
|  | 25-35 cases | 5 | 8.2 |
|  | 5-25 cases | 6 | 9.8 |
|  | less than 5 cases | 3 | 4.9 |
|  | More than 35 cases | 3 | 4.9 |
| How they diagnosed patients with FGS? | Urinary sediment and stool analysis | 13 | 21.3 |
|  | By signs and symptoms | 4 | 6.6 |
|  | Vaginal smear that revealed the presence of schistosome eggs | 1 | 1.6 |
|  | Examining the bleeding pictures of the cervix and hypogastralgia and then requested urinary sediment | 1 | 1.6 |
|  | The clinic that reveals the leucorrhea and urinary sediment that revealed the presence of schistosome eggs. | 1 | 1.6 |
|  | With vaginal touch | 1 | 1.6 |
| Diagnostic test availability | Yes | 35 | 57.4 |
|  | No | 25 | 41.0 |
| PZQ availability | Always | 21 | 34.4 |
|  | Once in a while | 10 | 16.4 |
|  | Sometimes | 30 | 49.2 |

**Test E**

Summary of FGS-like signs and symptoms experienced by the community women at Kifua II.

| **Variable** | **Response** | **Frequency** | **Percentage** |
| --- | --- | --- | --- |
| Are your menstruation periods irregular? | They are regular | 92 | 45.8 |
|  | Sometimes | 53 | 26.4 |
|  | They are irregular every month | 8 | 4.0 |
| Is your menstruation painful? | Always very painful | 11 | 5.5 |
|  | Sometimes very painful | 58 | 28.9 |
|  | It is normal | 84 | 41.8 |
| Do you have pain when urinating? | Yes | 38 | 18.9 |
|  | No | 163 | 81.1 |
| Do you have difficulty urinating (urine does not come out easily)? | Yes | 28 | 13.9 |
| Do you have a sudden, uncontrollable and unexpected urge to urinate and can't hold back? Even when you cough, it comes out? | Yes | 7 | 3.5 |
| Do you see blood in your urine? | Yes | 17 | 8.5 |
| When was the last time you saw blood in your urine? | Sometime | 12 | 6.0 |
|  | Always | 2 | 1.0 |
|  | Once in a while | 3 | 1.5 |
| Do you sometimes have itching in your private parts? | Yes | 55 | 27.4 |
| How often do you experience this? | Once in a while | 45 | 22.4 |
|  | Frequently | 10 | 5.0 |
| Do you have a burning sensation in your private area? | yes | 14 | 7.0 |
| How often do you experience this? | Once in a while | 12 | 6.0 |
|  | Frequently | 2 | 1.0 |
| Do you feel swelling/mass in your private area? | I do not know | 16 | 8.0 |
|  | Yes | 7 | 3.5 |
| Do you have discharge from your vagina? | Yes | 80 | 39.8 |
| Does the discharge smell? | Yes | 13 | 6.5 |
| Do you think this is normal? | No | 11 | 5.5 |
|  | Yes | 1 | 0.5 |
| What is its colour? | White | 73 | 36.3 |
|  | Yellow | 4 | 2.0 |
|  | Grey | 3 | 1.5 |
| After sex, do you have discharge? | Yes | 6 | 3.0 |
| Does it smell bad? | Yes | 5 | 2.5 |
| After or during sex, do you have pain? | Yes | 22 | 10.9 |
| Do you have blood flow? | Yes | 4 | 2.0 |
| Have you had miscarriages/pregnancies that ended early? | Yes | 26 | 12.9 |
| How many miscarriages | One | 16 | 8.0 |
|  | Two | 5 | 2.5 |
|  | Three | 3 | 1.5 |
|  | Eleven | 2 | 1.0 |
